# Functional genomics screen identifies YAP1 as a key determinant to enhance treatment sensitivity in lung cancer cells

**DOI:** 10.18632/oncotarget.6721

**Published:** 2015-12-22

**Authors:** Haiying Cheng, Zhenfeng Zhang, Ruth Rodriguez-Barrueco, Alain Borczuk, Huijie Liu, Jiyang Yu, Jose M. Silva, Simon K. Cheng, Roman Perez-Soler, Balazs Halmos

**Affiliations:** ^1^ Department of Oncology, Albert Einstein College of Medicine of Yeshiva University/Montefiore Medical Center, Bronx, NY, USA; ^2^ Sun Yat-Sen University Cancer Center, State Key Laboratory of Oncology in South China, Collaborative Innovation Center for Cancer Medicine, Center of Medical Imaging and Image-Guided Therapy, Guangzhou, China; ^3^ Icahn School of Medicine at Mount Sinai, New York, NY, USA; ^4^ Department of Pathology, Weill Cornell University Medical Center, New York, NY, USA; ^5^ Department of Biomedical Informatics, Columbia University, New York, NY, USA; ^6^ Department of Radiation Oncology, Columbia University College of Physicians and Surgeons, New York, NY, USA; ^7^ Department of Precision Medicine, Oncology Research Unit, Pfizer Inc., Pearl River, NY, USA; ^8^ Division of Hematology/Oncology, Herbert Irving Comprehensive Cancer Center, New York Presbyterian Hospital-Columbia University Medical Center, New York, NY, USA

**Keywords:** lung cancer, RNAi screen, YAP1, platinum resistance, radiation

## Abstract

Survival for lung cancer patients remains dismal and is largely attributed to treatment resistance. To identify novel target genes the modulation of which could modify platinum resistance, we performed a high-throughput RNAi screen and identified Yes-associated protein (YAP1), a transcription coactivator and a known oncogene, as a potential actionable candidate. YAP1 ablation significantly improved sensitivities not only to cisplatin but also to ionizing radiation, both of which are DNA-damaging interventions, in non-small cell lung cancer (NSCLC) cells. Overall YAP1 was expressed in 75% of NSCLC specimens, whereas nuclear YAP1 which is the active form was present in 45% of 124 resected NSCLC. Interestingly, *EGFR*-mutated or *KRAS*-mutated NSCLC were associated with higher nuclear YAP1 staining in comparison to *EGFR/KRAS* wild-type. Relevantly, YAP1 downregulation improved sensitivity to erlotinib, an EGFR inhibitor. A pharmacological inhibitor of YAP1 signaling, verteporfin also synergized with cisplatin, radiation and erlotinib in NSCLC cells by potentiating cisplatin and radiation-related double-stranded breaks and decreasing expression of YAP1 and EGFR. Taken together, our study is the first to indicate the potential role of YAP1 as a common modulator of resistance mechanisms and a potential novel, actionable target that can improve responses to platinum, radiation and EGFR-targeted therapy in lung cancer.

## INTRODUCTION

Lung cancer is the leading cause of cancer-related deaths worldwide. The mainstay of treatment for most patients with advanced lung cancer remains platinum-based chemotherapy, while radiation plays a key role in the treatment of locally-advanced disease, many times given concurrently with platinum-based chemotherapy. Despite recent treatment advances, primary and acquired resistance to chemotherapy and radiation therapy still leads to a dismal survival, around 16% at 5 year [1]. Platinum compounds function by forming platinum-DNA adducts and subsequently damaging DNA and thereby leading to cell death. Platinum-induced DNA damage is mainly repaired by the nucleotide excision repair pathway (NER) [2, 3]. The ERCC1 (excision repair cross-complementation group 1) protein, a key component of NER, has been implicated to be an important factor in platinum resistance [2, 3]. However, even in ERCC1-low tumors, only half of the patients respond to platinum therapy and all tumors ultimately develop resistance over time. This suggests accordingly that the resistance mechanisms to platinum may be multifactorial and likely multiple elements remain to be further elucidated. Radiation causes DNA damage and cell death via the generation of free radicals. Enhanced DNA repair or damage tolerance have been implicated in radiation resistance [4]. Besides ERCC1, other factors involved in DNA repair pathways, such as BRCA1, BRCA2, and poly (ADP-ribose) polymerase (PARP) have also been implicated in platinum resistance [5–8]. Moreover, further epigenetic and genetic alterations, such as changes in BCL-2 family members and caspases, have been linked to regulation of platinum responses [6]. Nevertheless, there are no clinically proven biomarkers for either platinum or radiation response. On the other hand, clinical outcomes in subgroups of lung cancer patients have been improved by identifying and targeting key oncogenic driver mutations, such as activating *EGFR* mutations [9]. Nearly all tumors progress over time due to acquired drug-resistance to EGFR-targeted therapy [9–13] and two of the major molecular mechanisms of such resistance include secondary *EGFR* T790M mutations and *MET* amplification [9]. Nevertheless, the resistance mechanisms to anti-EGFR treatment remain unknown in about one third of the cases. Taken together, there is a definite need to identify predictive biomarkers for these commonly used modalities as well as to develop novel synergistic therapies based on better understanding of biology in order to improve on our current treatment paradigms in lung cancer.

To address these needs, we have completed a high-throughput RNAi screen to identify, in an unbiased fashion, modulating factors of platinum resistance and have identified YAP1, a transcription co-activator, as a novel actionable candidate to synergize with platinum therapy. Our studies further indicate that YAP1 may be a common modulator of resistance mechanisms to platinum, radiation and EGFR-targeted therapy. Both genetic and pharmacological YAP1 inhibition was associated with improved sensitivities to these treatment modalities in lung cancer cells.

## RESULTS

### High-throughput RNAi screen for cisplatin resistance and synergistic partners

In order to understand the pathways that can synergize with cisplatin, we utilized a high-throughput RNAi platform to screen for novel target genes the modulation of which could influence drug sensitivity of lung cancer cells in response to platinum. The RNAi high-throughput screen with the shRNA library used in our study has previously been applied in several cancer types and has led to the successful identification of multiple novel key targets [14, 15].

**Figure 1:**
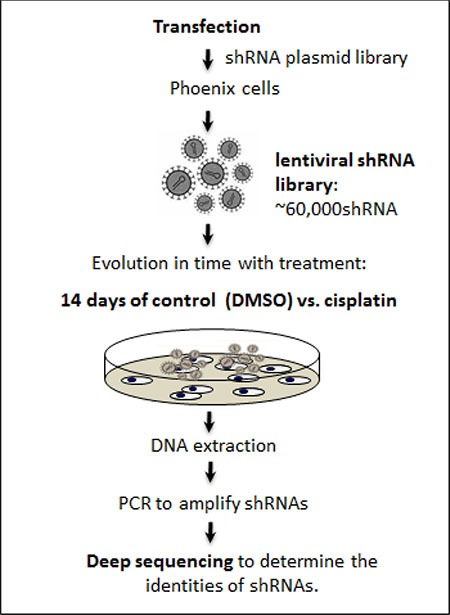
Schema of RNAi screen for cisplatin A pooled library of shRNA-mirs was introduced to lung cancer cells (PC9) at a multiplicity of infection of one (MOI = 1). Cells were treated for 14 days with control (DMSO vehicle) vs. cisplatin. Subsequently, genomic DNA was extracted, shRNAs were amplified, and deep sequencing was performed to determine the identities of shRNAs.

We first transfected lung cancer cells (PC9) with an shRNA viral library containing ~60,000 individual shRNAs targeting almost the entire human genome, and then identified depleted versus enriched shRNAs following two weeks of vehicle DMSO control vs. cisplatin treatment in surviving cells (Figure 1). We hypothesized that the shRNAs that were depleted more in cisplatin-treated versus control cells might represent genes whose function is key for cell survival in the face of cisplatin-induced damage. Thereby, inhibiting these genes could potentially synergize with cisplatin therapy. As previously published [14, 17, 18], we utilized a rigorous set of criteria and procedures for analyzing data, using the Bioconductor and Extraction of Differential Gene Expression libraries in R program. Standard quality control studies suggested excellent correlation between replicates. For instance, the Pearson correlation and Spearman correlation coefficients were 0.88 and 0.85 respectively for cisplatin triplicates. The genes from the initial positive “hits” screen were ranked as mentioned in the methods section. After using strict selection criteria, we selected a narrow list of highly depleted genes from the cisplatin screen, including Desmoglein 3 (−116 fold change), Aurora kinase A (−113 fold change), and YAP1 (Yes associated protein) (−80 fold change) (Table 1). Moreover, a preliminary and limited validation study revealed that siRNA targeting above three genes effectively silenced their expression and improved cisplatin sensitivity (data not shown).

**Table 1:** Top 20 primary screen hits in the cisplatin screen

Gene Symbol	Description	Fold Change	*P*-value	Validated in preliminary screen
**DHRS3**	Dehydrogenase/reductase (SDR family) member 3	−151.74	7.33E-04	
**DIP2B**	DIP2 disco-interacting protein 2 homolog B	−142.05	1.38E-04	
**HCN2, LOC100134399**	Hyperpolarization-activated, cyclic nucleotide-gated K+ 2	−138.49	8.43E-04	
**RRP1**	Recombination repair protein 1	−128.13	6.31E-04	
**PFKFB3**	6-phosphofructo-2-kinase/fructose-2, 6-biphosphatase 3	−125.69	1.03E-03	
**DSG3**	Desmoglein 3	−115.58	3.35E-04	Yes
**AURKA, AURKAPS1**	Aurora kinase A	−112.96	5.27E-03	Yes
**SMURF2**	SMAD specific E3 ubiquitin protein ligase 2	−82.51	2.65E-04	
**JAKMIP3**	Janus kinase and microtubule interacting protein 3	−81.4	5.28E-04	
**YAP1**	Yes-associated protein 1	−81.18	1.54E-03	Yes
**C6orf140 Or GLYATL3**	GLYATL3 glycine-N-acyltransferase-like 3	−79.83	1.88E-03	
**SLC35E3**	Solute carrier family 35, member E3	−79.1	4.19E-03	
**ATP1B2**	ATPase, Na+/K+ transporting, beta 2 polypeptide	−76.97	5.50E-04	
**CCDC9**	Coiled-coil domain containing 9	−76.73	3.57E-04	
**CAT**	Catalase	−73.97	3.01E-03	
**VARS2**	Valyl-tRNA synthetase 2, mitochondrial	−73.71	2.43E-04	
**LOC150577**	Long intergenic non-protein coding RNA 1104	−72.77	1.25E-03	
**FAM159A**	Family with sequence similarity 159, member A	−72.74	1.52E-03	
**OR10A3**	Olfactory receptor, family 10, subfamily A, member 3	−72.36	1.35E-03	

Fold change indicates relative fold depletion of shRNA primary screen hairpin hits in cisplatin vs. DMSO control

### YAP1 was identified as a potential candidate modulator of cisplatin resistance

**Figure 2:**
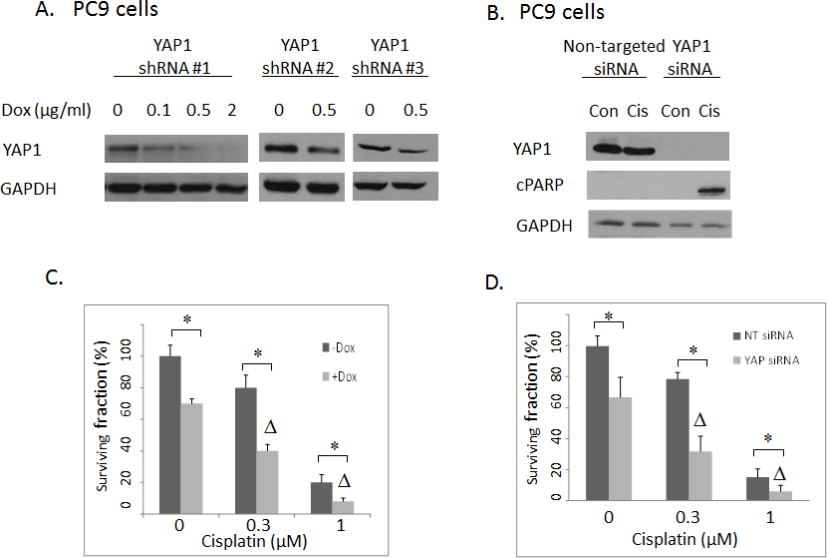
(**A**) Western blotting: YAP1 shRNA #1, #2 and #3 were introduced to PC9 cells separately with different concentrations of doxycycline (Dox) induction. YAP1 shRNA#1 had the highest knockdown efficiency and was used in the subsequent experiments. (**B**) Western blotting: The effects of YAP1 siRNA transfection to PC9 cells. cPARP: cleaved PARP. Con: Vehicle DMSO control. Cis: 1 uM cisplatin. (**C**) Clonogenic assay: The effects of inducible shRNA #1-mediated YAP1 knockdown on cisplatin activities in PC9 cells. Δ indicates that there was a synergistic effect between cisplatin and shRNA-mediated YAP1 knockdown (CI < 1). * = *P* < 0.05. –Dox: absence of doxycycline. + Dox: presence of 2 ug/ml doxycycline. (**D**) Clonogenic assay: The effects of siRNA-mediated YAP1 knockdown on cisplatin responses in PC9 lung cancer cells. Δ indicates that there was a synergistic effect between cisplatin and siRNA-mediated YAP1 knockdown (CI < 1). * = *P* < 0.05.

**Figure 3:**
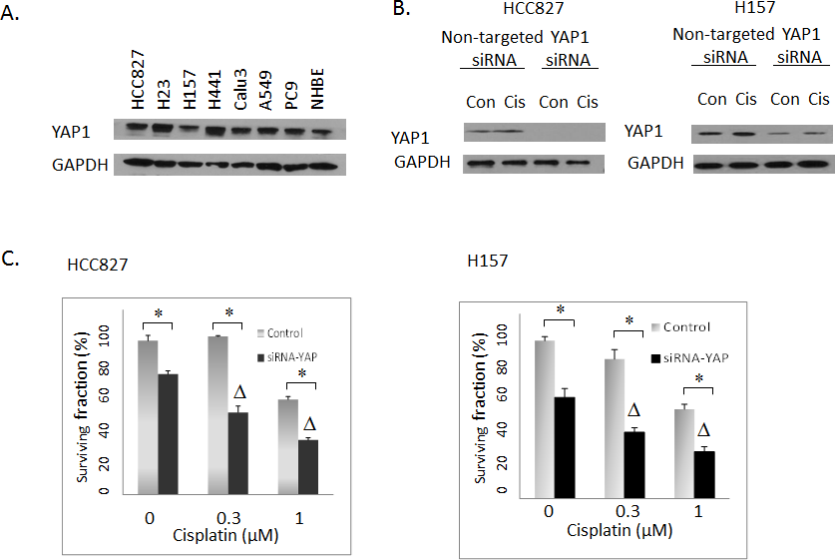
(**A**) Western blotting: Expression of YAP1 in NSCLC cell lines. NHBE: normal human bronchial epithelial cells. (**B**) Western blotting: The effects of YAP1 siRNA in HCC827 and H157 cells. Con: Vehicle DMSO control. Cis: 3uM cisplatin. (**C**) Clonogenic assay: The effects of siRNA-mediated YAP1 knockdown on cisplatin activities in HCC827 and H157 lung cancer cells. Control: Non-targeted siRNA. Δ indicates that there was a synergistic effect between cisplatin and siRNA-mediated YAP1 knockdown (CI < 1). * = *P* < 0.05.

Our preliminary validation study indicated that YAP1 may be a bona fide mediator of cisplatin sensitivity in lung cancer cells. YAP1 shRNAs were depleted 80 fold in the cisplatin screen. YAP1 was initially identified as a protein interacting with the non-receptor tyrosine kinase c-Yes [24] and is now considered as a nuclear effector of the Hippo pathway that promotes cell growth as a transcriptional co-activator [25]. Several lines of evidence suggested its role in predicting tumor progression and drug responses. For instance, overexpression of YAP1 could contribute to progression and poor prognosis of NSCLC [26], while knockdown of YAP1 sensitized ovarian cancer cells to cisplatin, erlotinib (EGFR Tyrosine kinase inhibitor) and S12 (survivin inhibitor) [27]. To further validate this promising target identified in our screen, we first confirmed that different shRNAs and an siRNA pool targeting YAP1 can efficiently knockdown YAP1 protein expression in the PC9 lung cancer cell line (Figure 2A and 2B). Next, the effects of YAP1 knockdown on drug responses were examined. YAP1 knockdown by either shRNA (Figure 2C) or siRNA (Figure 2D) indeed quite prominently improved sensitivities to cisplatin in PC9 lung cancer cells in a synergistic manner (CI < 1). Moreover, YAP1 knockdown combined with platinum therapy also induced apoptosis as measured by induction of PARP cleavage (Figure 2B). Expression of YAP1 was examined in several NSCLC cell lines (Figure 3A). Combination experiments between YAP1 knockdown and cisplatin were further investigated in two other NSCLC cell lines with YAP1 expression, HCC827 and H157. Similar synergistic effects were observed (Figure 3C and 3D). Interestingly, YAP1 ablation by itself led to decreased colony formation in all three cell lines examined. Taken together, YAP1 knockdown not only conferred a “single-agent” activity but also sensitized NSCLC cells to cisplatin.

### YAP1 inhibition sensitized lung cancer cells to radiation

**Figure 4:**
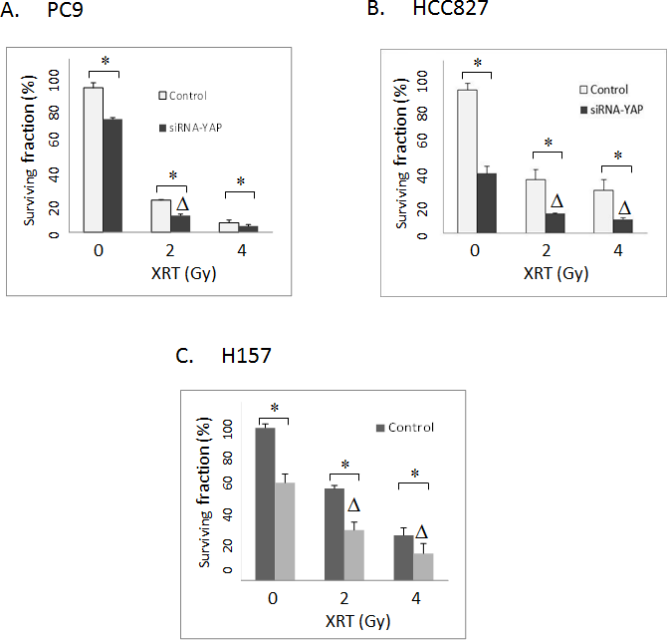
(**A**) Clonogenic assay: The effects of siRNA-mediated YAP1 knockdown on radiation in PC9 lung cancer cells. (**B**) Clonogenic assay: The effects of siRNA-mediated YAP1 knockdown on radiation in HCC827 lung cancer cells. (**C**) Clonogenic assay: The effects of siRNA-mediated YAP1 knockdown on radiation in H157 lung cancer cells. Control: Non-targeted siRNA. XRT: radiation. Δ indicates that there is a synergistic effect between radiation and siRNA-mediated YAP1 knockdown (CI < 1). * = *P* < 0.05.

Radiation treatment, like cisplatin, also causes DNA damage and can potentially share certain resistance mechanisms with cisplatin. Thus, we next examined the effects of knockdown of YAP1 on radiation sensitivities. As shown in Figure 4A and 4B, YAP1 knockdown synergized with radiation in PC9, HCC827 and H157 cells as determined by clonogenic assays (CI < 1, *P* < 0.05).

### YAP1 expression in primary human NSCLC

**Figure 5:**
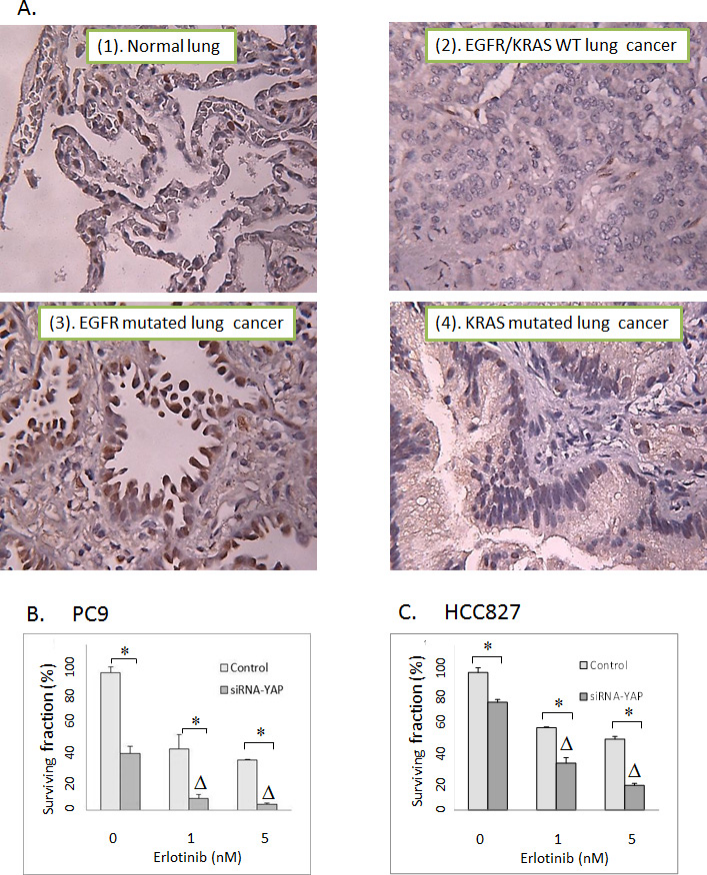
(A) Representative YAP1 IHC staining with tissue microarrays constructed with 124 surgically resected non-small cell lung tumors. (1). normal lung. (2). *EGFR/KRAS* wild type NSCLC. WT: Wild type. (3). *EGFR* mutated NSCLC. (4). *KRAS* mutated NSCLC. (**B**) Clonogenic assay: The effects of siRNA-mediated YAP1 knockdown on erlotinib response in PC9 lung cancer cells. (**C**) Clonogenic assay: The effects of siRNA-mediated YAP1 knockdown on erlotinib activity in HCC827 lung cancer cells. Control: Non-targeted siRNA. Δ indicates that there is a synergistic effect between erlotinib and siRNA-mediated YAP1 knockdown (CI < 1). * = *P* < 0.05.

To determine the expression profile of YAP1 in primary lung tumors, we performed IHC with YAP1 staining using tissue microarrays constructed from 124 well-annotated NSCLC tumor tissues (derived from stage I to III non-small cell lung cancer specimens from patients undergoing lung cancer resection between 2010 and 2012). Positive cases were defined as samples with positive YAP1 staining (either cytoplasmic or nuclear YAP1 or both). YAP1 was noted to be expressed in 77% of tumors. About half of the tumor specimens stained positive for nuclear YAP1, which is the functionally active form of YAP1 (Table 2). The nuclear YAP1 staining was positive in 50% of lung adenocarcinoma, 59% of squamous cell carcinoma and 32% of large cell carcinoma, respectively. Interestingly, among cases where *EGFR* and *KRAS* mutation status were known (*n* = 76), there was a higher YAP1 nuclear staining in *EGFR*-mutated (*n* = 16, average H score = 47, positive nuclear YAP1 in 63% of patients) or *KRAS*-mutated (*n* = 23, average H score = 51, positive nuclear YAP1 in 65% of patients) lung adenocarcinoma in comparison to *EGFR*/*KRAS* wild type (*n* = 37, average H score = 19, positive nuclear YAP1 in 43% of patients) (*P* < 0.05) (Figure 5, Table 2). A potential link between YAP1 and EGFR-RAS mediated oncogenesis was implicated by a recent observation that activation of EGFR-RAS signaling activated YAP1 and subsequently promoted glial cell growth [28]. Another study also indicated a positive correlation between YAP1 expression and EGFR mutations [29]. In concordance with these observations, our data suggest that EGFR/KRAS driven signaling might activate YAP1 leading to its nuclear translocation in lung cancers.

**Table 2:** YAP1 IHC staining with tissue microarrays constructed with 124 surgically removed tissues

	Positive nuclear YAP1 *n* (%)	Positive cytoplasmic YAP1 *n* (%)	Total positive YAP1 *n* (%)	Average H score of nuclear YAP1
All cases (*n* = 124)	58 (47%)	46 (37%)	95 (77%)	32
**Histology**				
Adeno (*n* = 74)	37 (50%)	32 (43%)	64 (86%)	34
Squamous (*n* = 22)	13 (59%)	6 (27%)	16 (73%)	41
Large cell (*n* = 19)	6 (32%)	5 (26%)	11 (53%)	20
Other (*n* = 9)	2 (22%)	3 (33%)	5 (56%)	23
**Mutation status**				
EGFR mutated (*n* = 16)	10 (63%)	7 (44%)	15 (94%)	47
KRAS mutated (*n* = 23)	15 (65%)	7 (30%)	20 (87%)	51
EGFR/KRAS wild type (*n* = 37)	16 (43%)	17 (46%)	21 (57%)	19

Positive cases were defined as samples with positive YAP1 staining (either cytoplasmic or nuclear YAP1 or both). In some cases, there was both nuclear and cytoplasmic YAP1 staining. The semi-quantitative H-score was determined as the sum of the percentage of staining (proportion score) multiplied by an ordinal value corresponding to staining intensity in the specimen (0 = none, 1 = weak, 2 = moderate, 3 = strong).

### YAP1 inhibition sensitized lung cancer cells to erlotinib

Our IHC experiments indicated that *EGFR*-mutated lung cancers were associated with higher YAP1 activation, thus YAP1-targeted therapy may potentiate the cytotoxicity of erlotinib (tyrosine kinase inhibitor targeting EGFR) in *EGFR*-mutated NSCLC cells. To test this possibility, we examined the effects of siRNA-mediated YAP1 knockdown on erlotinib cytotoxicity. As shown in Figure 5B and 5C, YAP1 blockage indeed synergized with erlotinib in both PC9 and HCC827 cells (CI < 1, *P* < 0.05).

### Verteporfin, a pharmacological inhibitor of YAP1, sensitized NSCLC cells to cisplatin, radiation and erlotinib

**Figure 6:**
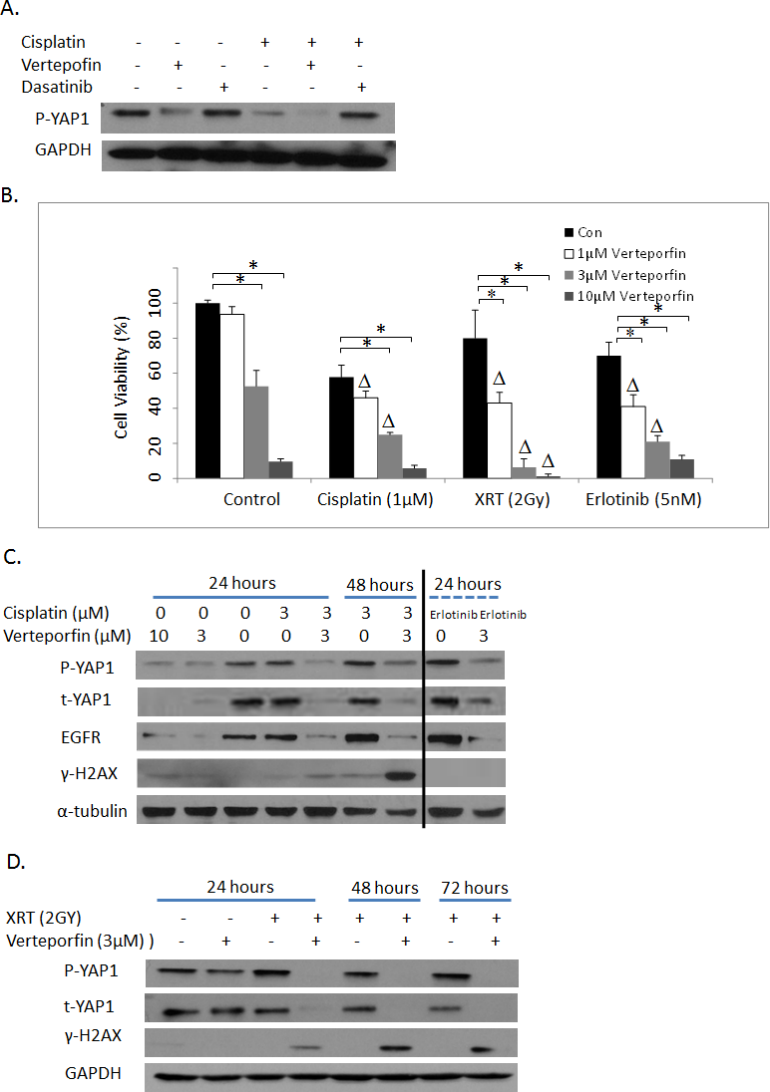
(**A**) Western blotting: The effects of verteporfin and dasatinib on YAP1 phosphorylation in PC9 cells. (**B**). MTS assay: The effects of verteporfin on cytotoxicities of cisplatin, XRT and erlotinib in PC9 lung cancer cells. Con: Vehicle DMSO control. XRT: radiation. Δ indicates that there is a synergistic effect between verteporfin and cisplatin, XRT and erlotinib (CI < 1). * = *P* < 0.05. (**C**) Western blotting: The effects of verteporfin and cisplatin (first 7 lanes) on YAP1 and γ-H2AX in PC9 cells. The last two lanes indicated the effects of verteporfin and erlotinib on YAP1 and γ-H2AX. (**D**) Western blotting: The effects of verteporfin and XRT on YAP1 and γ-H2AX in PC9 cells. p-YAP1: phosphos-YAP1; t-YAP1: total-YAP1.

We further examined the effects of a pharmacological inhibitor of YAP1 signaling, verteporfin, on cytotoxicity of cisplatin, radiation and erlotinib. The association of YAP1 and TEAD transcription factor has been reported to be essential for YAP1-driven oncogenic growth. A recent study suggested that verteporfin could disrupt the active YAP1-TEAD complex and thereby leads to inhibition of YAP1-induced liver overgrowth [30]. Verteporfin is currently used in clinic for the treatment of neovascular macular degeneration. As shown in Figure 6A, verteporfin indeed reduces phosphorylation of YAP1 in PC9 lung cancer cells at baseline or in the presence of cisplatin, whereas a YES inhibitor, dasatinib had no such effects. Moreover, verteporfin treatment improved cisplatin cytotoxicity synergistically (Figure 6B, CI < 1, *P* < 0.05). Verteporfin also synergizes with radiation and erlotinib in PC9 lung cancer cells suggestive of a functional synergism upon YAP1 blockade (Figure 6B, CI < 1, *P* < 0.05). To gain insights into the underlying mechanisms of the synergism between verteporfin and cisplatin, we next investigated their effects on γ-H2AX, induction of which is considered as a marker for DNA double strand breaks (DSBs). As shown in Figure 6C, there were persistent and more pronounced γ-H2AX induction with combined verteporfin and cisplatin treatment than either agent alone, especially at 48 hours. Similarly, sustained and marked γ-H2AX levels were also noticed with combined verteporfin and radiation, even though these agents used alone at low dose only led to baseline or minimal γ-H2AX induction (Figure 6C and 6D). Furthermore, consistent with a recent study in esophageal cancer [31], verteporfin diminished both total and phospho-YAP1 levels and EGFR expression, both alone and in combination with cisplatin or erlotinib or radiation treatment. Thus, verteporfin can influence EGFR signaling by down-regulating EGFR expression. Similar effects of verteporfin on another NSCLC cell line, HCC827 were also observed (data not shown). These data indicate that verteporfin can overcome resistance and sensitize NSCLC cells to cisplatin and radiation by at least two means: increasing levels of DSBs (more γ-H2AX) and decreasing expression of YAP1.

## DISCUSSION

Platinum-based chemotherapy and chemoradiotherapy are the mainstay of the treatment paradigms for stage III/IV NSCLC. Nevertheless, platinum and radioresistance remain pressing clinical problems. The underlying mechanisms are likely multi-factorial and have remained largely elusive. In the present study, we performed a genome-wide high throughput RNAi screen to search for novel synergistic targets the modulation of which could influence platinum sensitivity in lung cancer cells. We first identified YAP1 as a potential lead candidate to affect platinum sensitivity. YAP1 shRNAs were significantly depleted at 80 fold in the cisplatin RNAi screen. We then showed that both genetic (shRNA or siRNA) and pharmacological YAP1 blockade (verteporfin) improved cisplatin activity in lung cancer cells. Additionally, YAP1 inhibition also synergized with radiation and the EGFR TKI, erlotinib. By targeting YAP1 signaling, verteporfin potentiated cisplatin and radiation-related DSBs and decreased expression of YAP1 and EGFR. Thus, our data suggests that YAP1 may be a common modulator of resistance mechanisms to platinum, radiation and EGFR-targeted therapies in lung cancer.

As a transcriptional coactivator, YAP1 is the downstream effector of the Hippo signaling pathway which is an essential regulator of organ size and tumor growth by modulating cell proliferation and apoptosis [32]. Culminating evidence suggest that YAP1 is involved in the modulation of cancer cell proliferation, self-renewal of cancer stem cells and initiation of migration and metastasis [32, 33]. YAP1 expression has been suggested as a negative prognostic factor for lung cancer survival [26]. Under nonproliferating conditions, YAP1 is phosphorylated and retained in the cytoplasm. Upon activation, YAP1 accumulates in the nucleus, and then associates with transcription factors, such as TEAD, to regulate the expression of genes that promote cell proliferation [32, 34, 35]. Our study showed that total YAP1 was expressed in 75% and the active form of YAP1, nuclear YAP1 was expressed in 48% of primary lung cancer specimens which is in line with previous report that YAP1 was expressed in 66% of cases and is found to be present predominantly in nucleus [26]. Our data also demonstrated that *EGFR* mutated or *KRAS* mutated lung cancers are associated with higher nuclear YAP1 expression in comparison to *EGFR/KRAS* wild type tumors. A recent study identified YAP1 as essential KRAS effector in the development of pancreatic ductal adenocarcinoma [36]. The results also corroborate prior findings suggestive of YAP1 expression to be regulated by oncogenic EGFR/KRAS signaling [28] and higher YAP1 expression in tumors with *EGFR* mutations [29].

Moreover, YAP1 has been implicated in the modulation of drug sensitivities. For instance, YAP1 was shown to affect activities of cisplatin and EGFR inhibitors in ovarian cancer cells [27]. In the context of radiotherapy, YAP1 expression was predictive of radio-resistance in patients with head and neck cancer [37]. YAP1 has also been indicated in mediating resistance to anti-tubulin drugs, such as paclitaxel, in a Hippo-independent manner [38]. A recent study utilizing genetic screen further identified YAP1 as a resistance mechanism to RAF- and MEK-targeted therapies [17]. In accordance with previous findings, YAP1 was identified in our RNAi screen as a potential synergistic target for cisplatin. YAP1 knockdown potentiated the effects of cisplatin, and also the activities of radiation. Both cisplatin and radiation can lead to DNA damage with accumulation of DNA double strand breaks. Our study showed that the combination of verteporfin (YAP1 inhibitor) along with either cisplatin or radiation led to persistent γ-H2AX formation (marker for DNA double strand breaks) which is suggestive of continuous and unrepaired DNA damages caused by the combination. Thus, down-regulation of YAP1 can sensitize cells to DNA damaging agents.

Several studies indicated that pharmacological YAP1 inhibitors including verteporfin could suppress tumor growth or sensitize tumor cells to cytotoxics in a variety of tumor types, such as esophageal cancer, rhabdomyosarcoma, ovarian cancer and bladder cancer [19, 31, 39, 40]. Our data also indicate that YAP1-targeted therapy, verteporfin, diminished EGFR expression and was associated with better erlotinib cytotoxicity. The interplay between YAP1 and EGFR signaling was noted previously where EGFR activation induced YAP1 gene expression in a study of hepatocellular carcinoma [41], while YAP1 induced EGFR expression in another report [31]. Furthermore, a study indicated that the expression of cytoplasmic YAP1, which is the inactive form, was associated with longer-progression free survival and overall survival in EGFR-TKI-treated lung cancer patients with *EGFR* mutations [42]. Thereby, YAP1 blocking agents may also help to improve EGFR-targeted therapy.

In summary, the present study provides evidence to identify YAP1 as a key and actionable determinant of sensitivities to platinum, radiation and erlotinib in lung cancer cells. We have shown that YAP1 blockade could sensitize lung cancer cells to cisplatin, radiation and erlotinib. This observation may have very important clinical implications as it sets up the foundation to utilize YAP1-targeted modalities in combination with other cancer therapies to achieve better therapeutic outcomes, especially in YAP1-positive lung cancers. Future mechanistic studies are certainly needed to demonstrate how exactly YAP1 functions as a common determinant modifying such diverse resistance mechanisms.

## MATERIALS AND METHODS

### Cell lines and material

The following NSCLC cell lines were obtained from American Type Tissue Collection (Manassas, VA): HCC827, H23, H157, H441, A549 and NHBE (normal human bronchial epithelial cells). PC9 cells were a gift from Dr. Susumu Kobayashi, Harvard Medical School, Boston, MA. Lung cancer cells were grown in RPMI 1640 supplemented with 10% FBS and 1x Antibiotic/Antimycotic (Invitrogen, Carlsbad, CA) and were in the logarithmic growth phase at the initiation of all experiments. Verteporfin and Dasatinib (BMS-354825) were obtained from Selleck Chemicals (Houston, TX); Verteporfin is a photosensitizer, thus we conducted all experiments involving verteporfin in the darkness. The cell cultures with verteporfin were protected from ambient light. Cisplatin was obtained from Sigma-Aldrich (St. Louis, MO).

### High throughput RNAi screen

As previously described [14–17] (Figure 1), we first transfected lung cancer cells (PC9) with an shRNA viral library containing 58,493 shRNA-mirs in a pooled format at a multiplicity of infection of one (MOI = 1). Cells were then treated for 14 days with DMSO as vehicle control vs. cisplatin (1 μM). The transduced cells were plated in triplicates under each experimental condition. Subsequently, genomic DNA was extracted, shRNAs were amplified, and next generation sequencing was performed at the Columbia University Genomic Center to determine the identities of shRNAs. As previously published [14, 17, 18], we utilized a rigorous set of criteria and procedures for analyzing the derived data, using the Bioconductor and Extraction of Differential Gene Expression libraries in R program. Correlation between biological replicates was calculated. The genes from the initial positive “hits” screen were ranked based on the following criteria: 1. the presence of multiple shRNAs targeting the same gene; 2. the strength of the phenotype (fold change in representation). Given that a large number of hits on the primary screen were statistically significant (*P* < 0.05), we further classified these candidates based on: 1. 50-fold or greater depletion of corresponding shRNA (~top 3% of the primary hits with *P* < 0.01); 2. established expression in lung cancer with known function in carcinogenesis or chemotherapy resistance.

### Immunoblotting and antibodies

Proteins were separated by 10% SDS-PAGE gel and transferred onto PVDF membranes (Bio-Rad) for Western blot analysis as described previously [19]. The antibody against YAP1 (H-125) was purchased from Santa Cruz Biotechnology (Santa Cruz, CA). Antibodies against Phospho-YAP1 (Ser127), Cleaved PARP (Asp214), tubulin and Glyceraldehyde-3-phosphate dehydrogenase (GAPDH) were purchased from Cell Signaling Technology (Boston, MA). Antibody against phosphor-Histone H2AX (Ser139) was purchased from Millipore (Temecula, CA).

### Immunohistochemistry

As previously described [19], formalin-fixed primary lung tumor tissue sections were deparaffinized followed by antigen retrieval treatment with sodium citrate (10 mM, pH 6.0). Endogenous peroxidase activity was blocked by Dako peroxidase blocking reagent (Dako Corporation, Carpinteria, CA). YAP1 (H125) antibody was used at a dilution of 1:1000 (optimal dilution for overnight incubation) at room temperature. Then DakoCytomation LSAB 2 system-HRP (Dako Corporation, Carpinteria, CA) was used by adding biotinylated link universal and streptavidin-HRP followed by DAB chromogen (Dako Corporation, Carpinteria, CA) and hematoxylin nuclear counterstaining. The expression of YAP1 was evaluated by using immunohistochemistry (IHC) in FFPE patients' specimens by two independent investigators and its immunohistochemical semi-quantitation was performed using the H-score. Positive cases were defined as samples with positive YAP1 staining (either cytoplasmic or nuclear YAP1 or both). The H-score was determined as the percentage of staining (proportion score) multiplied by an ordinal value corresponding to the maximum intensity level in the specimen (0 = none, 1 = weak, 2 = moderate, 3 = strong).

### Drug combination studies with clonogenic survival assay

Clonogenic survival was assayed as previously described [20]. Logarithmically growing cells were plated in triplicate in 6-well tissue culture dishes containing media alone (200 cells per well) or media supplemented with drugs (including radiation) (500 cells per well). The plating efficiency (PE) was around 50% for untreated controls. Cisplatin and erlotinib were added for 3 days [5]. After 7–14 days, colonies were fixed with 70% EtOH and stained with 0.5% crystal violet. Surviving colonies were defined as colonies containing > 50 cells. Survival was expressed as the relative plating efficiency as compared to control cells. The Bliss Model was used to calculate the combination index (CI) and to evaluate the effects of drug combinations [21]. f1, f2 and f12 represents the effects from single drug 1, single drug 2 and the drug combination of drugs 1 & 2. The Bliss combination index (CI_Bliss_) = (f1 + f2 − f1 × f2)/f12. The drug-drug interactions (or drug − YAP1 knockdown interaction) were defined as synergism if CI < 1, antagonism if CI > 1, otherwise additive. CIs were also checked by Calcusyn software (Biosoft) based on the method of Chou and Talalay [22]. Similar CI trends were observed with both methods.

### MTS cell growth assay

Lung cancer cells were seeded at a density of 3000 cells (or 1500 cells for siRNA experiments) per well in 96-well plates in RPMI 1640 containing 10% FBS on day 0. Increasing concentrations of cisplatin (0.1 μM, 1 μM) or radiation (2 Gy, 4 Gy) or erlotinib (1 nM, 5 nM) were introduced on day 1 with or without YAP1 inhibition (either genetically or pharmacologically with verteporfin) [23]. The cells were incubated in the presence of drug treatment for 3 days. Viable cell numbers on day 4 were determined using the MTS assay kit according to the manufacturer's protocol (Promega, Madison, WI). Each assay consisted of three replicate wells.

### Small interfering RNA (siRNA) knockdown

Knockdown of YAP1 was performed using siRNA pools targeting YAP1 (ON-TARGET plus SMARTpool) purchased from Dharmacon RNAi Technologies (Thermo, Rockford, IL). SiGENOME Nontargeting siRNA Pools served as negative control. Introduction of siRNA (300 nM) was performed with DharmaFect1 transfection reagent according to the manufacturer's instructions (Thermo). Levels of YAP1 knockdown at different time points were assessed by immunoblot analysis from pools of transfected cells.

### Inducible shRNA knockdown

The Thermo Scientific Open Biosystems Expression Arrest TRIPZ Lentiviral shRNAmir was used for inducible shRNA knockdown of YAP1 per manufacturer's manual (TRIPZ Human YAP1 shRNA, Thermo Fisher Scientific, PA, USA). Individual TRIPZ Human YAP1 shRNA (Clone ID: V2THS_247011) was used as shRNA #1, TRIPZ Human YAP1 shRNA (Clone ID: V2THS_65508) was used as shRNA #2 and TRIPZ Human YAP1 shRNA (Clone ID: V2THS_65509) was used as shRNA#3. Doxycycline induction led to efficient knockdown of YAP1 as assessed by Western blotting. Pooled NSCLC cells with inducible shRNA-YAP1 were only utilized within a month following transduction.

### Statistics

All data are expressed as means ± SD from at least triplicate experiments. Statistical analysis of cell lines was performed by one- or two-way ANOVA, as appropriate using Statistica 6.0 (StatSoft, Tulsa, OK). YAP1 expression levels by IHC were compared between groups defined by mutation status and histology with the Kruskal-Wallis test. A two-sided *p*-value < 0.05 was considered statistically significant.

## References

[1] Siegel R, Naishadham D, Jemal A (2012). Cancer statistics 2012. CA Cancer J Clin.

[2] Wang D, Lippard SJ (2005). Cellular processing of platinum anticancer drugs. Nat Rev Drug Discov.

[3] Olaussen KA, Dunant A, Fouret P, Brambilla E, Andre F, Haddad V, Taranchon E, Filipits M, Pirker R, Popper HH, Stahel R, Sabatier L, Pignon JP (2006). DNA repair by ERCC1 in non-small-cell lung cancer and cisplatin-based adjuvant chemotherapy. N Engl J Med.

[4] Parplys AC, Petermann E, Petersen C, Dikomey E, Borgmann K (2012). DNA damage by X-rays and their impact on replication processes. Radiother Oncol: journal of the European Society for Therapeutic Radiology and Oncology.

[5] Cheng H, Zhang Z, Borczuk A, Powell CA, Balajee AS, Lieberman HB, Halmos B (2013). PARP inhibition selectively increases sensitivity to cisplatin in ERCC1-low non-small cell lung cancer cells. Carcinogenesis.

[6] Galluzzi L, Vitale I, Michels J, Brenner C, Szabadkai G, Harel-Bellan A, Castedo M, Kroemer G (2014). Systems biology of cisplatin resistance: past, present and future. Cell Death Dis.

[7] Papadaki C, Sfakianaki M, Ioannidis G, Lagoudaki E, Trypaki M, Tryfonidis K, Mavroudis D, Stathopoulos E, Georgoulias V, Souglakos J (2012). ERCC1 and BRAC1 mRNA expression levels in the primary tumor could predict the effectiveness of the second-line cisplatin-based chemotherapy in pretreated patients with metastatic non-small cell lung cancer. J Thorac Oncol.

[8] Michels J, Vitale I, Galluzzi L, Adam J, Olaussen KA, Kepp O, Senovilla L, Talhaoui I, Guegan J, Enot DP, Talbot M, Robin A, Girard P (2013). Cisplatin resistance associated with PARP hyperactivation. Cancer Res.

[9] Pao W, Hutchinson KE (2012). Chipping away at the lung cancer genome. Nat Med.

[10] Sequist LV, Waltman BA, Dias-Santagata D, Digumarthy S, Turke AB, Fidias P, Bergethon K, Shaw AT, Gettinger S, Cosper AK, Akhavanfard S, Heist RS, Temel J (2011). Genotypic and histological evolution of lung cancers acquiring resistance to EGFR inhibitors. Sci Transl Med.

[11] Engelman JA, Zejnullahu K, Mitsudomi T, Song Y, Hyland C, Park JO, Lindeman N, Gale CM, Zhao X, Christensen J, Kosaka T, Holmes AJ, Rogers AM (2007). MET amplification leads to gefitinib resistance in lung cancer by activating ERBB3 signaling. Science.

[12] Takezawa K, Pirazzoli V, Arcila ME, Nebhan CA, Song X, de Stanchina E, Ohashi K, Janjigian YY, Spitzler PJ, Melnick MA, Riely GJ, Kris MG, Miller VA (2012). HER2 amplification: a potential mechanism of acquired resistance to EGFR inhibition in EGFR-mutant lung cancers that lack the second-site EGFRT790M mutation. Cancer Discov.

[13] Zhang Z, Lee JC, Lin L, Olivas V, Au V, LaFramboise T, Abdel-Rahman M, Wang X, Levine AD, Rho JK, Choi YJ, Choi CM, Kim SW (2012). Activation of the AXL kinase causes resistance to EGFR-targeted therapy in lung cancer. Nat Genet.

[14] Silva JM, Marran K, Parker JS, Silva J, Golding M, Schlabach MR, Elledge SJ, Hannon GJ, Chang K (2008). Profiling essential genes in human mammary cells by multiplex RNAi screening. Science.

[15] Bivona TG, Hieronymus H, Parker J, Chang K, Taron M, Rosell R, Moonsamy P, Dahlman K, Miller VA, Costa C, Hannon G, Sawyers CL (2011). FAS and NF-kappaB signalling modulate dependence of lung cancers on mutant EGFR. Nature.

[16] Rodriguez-Barrueco R, Marshall N, Silva JM (2013). Pooled shRNA screenings: experimental approach. Methods Mol Biol.

[17] Lin L, Sabnis AJ, Chan E, Olivas V, Cade L, Pazarentzos E, Asthana S, Neel D, Yan JJ, Lu X, Pham L, Wang MM, Karachaliou N (2015). The Hippo effector YAP promotes resistance to RAF- and MEK-targeted cancer therapies. Nat Genet.

[18] Yu J, Putcha P, Califano A, Silva JM (2013). Pooled shRNA screenings: computational analysis. Methods Mol Biol.

[19] Ciamporcero E, Shen H, Ramakrishnan S, Yu Ku S, Chintala S, Shen L, Adelaiye R, Miles KM, Ullio C, Pizzimenti S, Daga M, Azabdaftari G, Attwood K (2015). YAP activation protects urothelial cell carcinoma from treatment-induced DNA damage. Oncogene.

[20] Franken NA, Rodermond HM, Stap J, Haveman J, van Bree C (2006). Clonogenic assay of cells *in vitro*. Nat Protoc.

[21] Peng H, Wen J, Li H, Chang J, Zhou X (2011). Drug inhibition profile prediction for NFkappaB pathway in multiple myeloma. PLoS One.

[22] Chou TC (2008). Preclinical versus clinical drug combination studies. Leukemia and lymphoma.

[23] Evers B, Drost R, Schut E, de Bruin M, van der Burg E, Derksen PW, Holstege H, Liu X, van Drunen E, Beverloo HB, Smith GC, Martin NM, Lau A (2008). Selective inhibition of BRCA2-deficient mammary tumor cell growth by AZD2281 and cisplatin. Clin Cancer Res.

[24] Sudol M (1994). Yes-associated protein (YAP65) is a proline-rich phosphoprotein that binds to the SH3 domain of the Yes proto-oncogene product. Oncogene.

[25] Bertini E, Oka T, Sudol M, Strano S, Blandino G (2009). YAP: at the crossroad between transformation and tumor suppression. Cell cycle.

[26] Wang Y, Dong Q, Zhang Q, Li Z, Wang E, Qiu X (2010). Overexpression of yes-associated protein contributes to progression and poor prognosis of non-small-cell lung cancer. Cancer science.

[27] Huang JM, Nagatomo I, Suzuki E, Mizuno T, Kumagai T, Berezov A, Zhang H, Karlan B, Greene MI, Wang Q (2013). YAP modifies cancer cell sensitivity to EGFR and survivin inhibitors and is negatively regulated by the non-receptor type protein tyrosine phosphatase 14. Oncogene.

[28] Reddy BV, Irvine KD (2013). Regulation of Hippo signaling by EGFR-MAPK signaling through Ajuba family proteins. Developmental cell.

[29] Gao Y, Zhang W, Han X, Li F, Wang X, Wang R, Fang Z, Tong X, Yao S, Li F, Feng Y, Sun Y, Hou Y (2014). YAP inhibits squamous transdifferentiation of Lkb1-deficient lung adenocarcinoma through ZEB2-dependent DNp63 repression. Nature communications.

[30] Liu-Chittenden Y, Huang B, Shim JS, Chen Q, Lee SJ, Anders RA, Liu JO, Pan D (2012). Genetic and pharmacological disruption of the TEAD-YAP complex suppresses the oncogenic activity of YAP. Genes and development.

[31] Song S, Honjo S, Jin J, Chang SS, Scott AW, Chen Q, Kalhor N, Correa AM, Hofstetter WL, Albarracin CT, Wu TT, Johnson RL, Hung MC (2015). The Hippo Coactivator YAP1 Mediates EGFR Overexpression and Confers Chemoresistance in Esophageal Cancer. Clin Cancer Res.

[32] Johnson R, Halder G (2014). The two faces of Hippo: targeting the Hippo pathway for regenerative medicine and cancer treatment. Nature reviews. Drug discovery.

[33] Piccolo S, Cordenonsi M, Dupont S (2013). Molecular pathways: YAP and TAZ take center stage in organ growth and tumorigenesis. Clinical cancer research.

[34] Pan D (2010). The hippo signaling pathway in development and cancer. Developmental cell.

[35] Staley BK, Irvine KD (2012). Hippo signaling in Drosophila: recent advances and insights. Developmental dynamics.

[36] Zhang W, Nandakumar N, Shi Y, Manzano M, Smith A, Graham G, Gupta S, Vietsch EE, Laughlin SZ, Wadhwa M, Chetram M, Joshi M, Wang F (2014). Downstream of Mutant KRAS, the Transcription Regulator YAP Is Essential for Neoplastic Progression to Pancreatic Ductal Adenocarcinoma. Science signaling.

[37] Akervall J, Nandalur S, Zhang J, Qian CN, Goldstein N, Gyllerup P, Gardinger Y, Alm J, Lorenc K, Nilsson K, Resau J, Wilson G, Teh B (2014). A novel panel of biomarkers predicts radioresistance in patients with squamous cell carcinoma of the head and neck. Eur J Cancer.

[38] Zhao Y, Khanal P, Savage P, She YM, Cyr TD, Yang X (2014). YAP-induced resistance of cancer cells to antitubulin drugs is modulated by a Hippo-independent pathway. Cancer Res.

[39] Hua G, Lv X, He C, Remmenga SW, Rodabough KJ, Dong J, Yang L, Lele SM, Yang P, Zhou J, Karst A, Drapkin RI, Davis JS (2016). YAP induces high-grade serous carcinoma in fallopian tube secretory epithelial cells. Oncogene.

[40] Slemmons K K, Crose LE, Rudzinski E, Bentley RC, Linardic CM (2015). Role of the YAP Oncoprotein in Priming Ras-Driven Rhabdomyosarcoma. PloS one.

[41] Urtasun R, Latasa MU, Demartis MI, Balzani S, Goni S, Garcia-Irigoyen O, Elizalde M, Azcona M, Pascale RM, Feo F, Bioulac-Sage P, Balabaud C, Muntane J (2011). Connective tissue growth factor autocriny in human hepatocellular carcinoma: oncogenic role and regulation by epidermal growth factor receptor/yes-associated protein-mediated activation. Hepatology.

[42] Sun PL, Kim JE, Yoo SB, Kim H, Jin Y, Jheon S, Kim K, Lee CT, Chung JH (2014). Cytoplasmic YAP Expression is Associated with Prolonged Survival in Patients with Lung Adenocarcinomas and Epidermal Growth Factor Receptor Tyrosine Kinase Inhibitor Treatment. Ann Surg Oncol.

